# Cardiovascular safety of 5-fluorouracil and capecitabine in colorectal cancer patients: real-world evidence

**DOI:** 10.1186/s40959-024-00294-2

**Published:** 2025-01-15

**Authors:** Chun-Ka Wong, Isaac Ho, Ali Choo, Rachel Lau, Ting-Fung Ma, Alston Conrad Ho-On Chiu, Tsun-Ho Lam, Minqing Lin, Ricky Wang-Hei Leung, Frankie Chor-Cheung Tam, Dominic Chi Chung Foo, Hung-Fat Tse

**Affiliations:** 1 Department of Medicine, School of Clinical Medicine, Li Ka Shing Faculty of Medicine, The University of Hong Kong, Hong Kong SAR, China; 2 Department of Clinical Oncology, Queen Mary Hospital, Hong Kong SAR, China; 3 Department of Statistics, University of South Carolina, Columbia, SC USA; 4 Department of Surgery, School of Clinical Medicine, Li Ka Shing Faculty of Medicine, The University of Hong Kong, Hong Kong SAR, China; 5 Cardiac and Vascular Center, Hong Kong University Shenzhen Hospital, Shenzhen, 518053 China; 6 Center for Translational Stem Cell Biology, Hong Kong SAR, China; 7 Department of Medicine, Queen Mary Hospital, The University of Hong Kong, Hong Kong SAR, China

**Keywords:** Colorectal carcinoma, Fluoropyrimidine, 5-fluorouracil, Capecitabine, MACE

## Abstract

**Background:**

Fluoropyrimidines, including 5-fluorouracil and capecitabine, are the most common chemotherapeutic agents for colorectal carcinoma. Although previous studies have suggested varying degrees of cardiotoxicity with these drugs, there is a notable lack of large-scale investigations with appropriate control groups. This study aimed to evaluate cardiovascular outcome among colorectal carcinoma patients treated with fluoropyrimidines.

**Methods:**

A retrospective propensity score- matched cohort study was conducted in patients diagnosed with colorectal carcinoma between January 1, 1993 and December 31, 2021 at public hospitals in Hong Kong. Cardiovascular outcomes in patients prescribed fluoropyrimidines were compared with controls. Further analyses to compare 5-fluroracil and capecitabine were performed.

**Results:**

A total of 51,888 colorectal carcinoma patients were identified. After 1:1 propensity score matching, 21,216 patients were included in the final analysis, with 10,608 patients in each group. 1.06% patients experienced a major adverse cardiovascular event (MACE) at 1 year. There was no significant difference in MACE risk between the two groups (HR 0.91, 95% confidence interval (95%CI): 0.70–1.18, *p* = 0.46). Risk of cardiovascular death was similar between the two groups (HR 1.05, 95%CI: 0.69–1.60, *p* = 0.82). Subgroup analysis did not demonstrate a statistically significant elevated risk of MACE during fluoropyrimidine use in high-risk patient groups. Further comparison of 5-fluorouracil and capecitabine did not reveal a difference in MACE (0.80% vs. 0.98%; HR 1.09, 95%CI: 0.64–1.85, *p* < 0.75).

**Conclusion:**

Fluoropyrimidine use in patients with colorectal carcinoma did not increase the risk of MACE, cardiovascular death, or other specific cardiovascular conditions. There was no significant difference in cardiovascular risk between 5-fluorouracil and capecitabine.

**Graphical Abstract:**

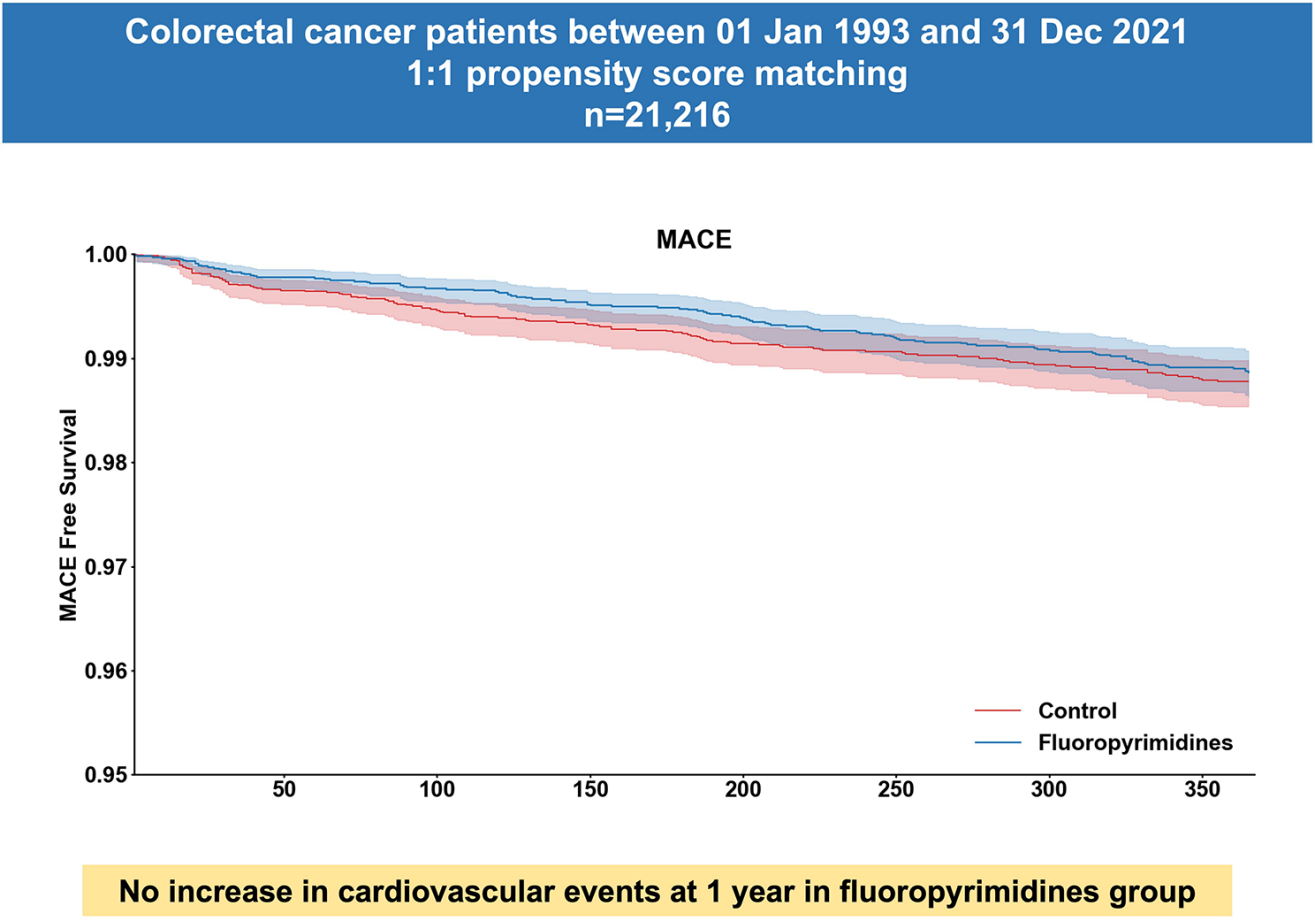

**Supplementary Information:**

The online version contains supplementary material available at 10.1186/s40959-024-00294-2.

## Introduction

Colorectal cancer is one of the most common cancers worldwide and causes a significant healthcare burden. Fluoropyrimidines are chemotherapeutic agents that form the backbone of colorectal cancer treatment. The two most common fluoropyrimidines are 5-fluorouracil, administered parenterally, and its oral prodrug capecitabine. These agents have been shown to enhance overall survival and decrease tumor recurrence in colorectal cancer patients [1, 2]. The choice of 5-fluorouracil or capecitabine is determined by multiple well-recognised factors that include their distinct route of administration, varying demand on renal function, and disparate toxicity profiles, such as diarrhea, vomiting, and palmar-plantar erythrodysesthesia [3].

Fluropyrimidines are associated with a range of cardiovascular events. The most common manifestations are angina and electrocardiograhic changes [4, 5]. Major cardiovascular events such as myocardial infarction, ischemic stroke, and arrhythmia have been reported in post-marketing case reports or uncontrolled retrospective observational studies [6–8]. Recent studies with matched non-cancer controls showed that patients with gastrointestinal cancer and no prior ischemic heart disease treated with 5-fluorouracil had a higher risk of myocardial infarction, albeit with a low overall incidence of 0.9% [9]. Nonetheless interpreting such real-world data using non-cancer patients as controls is challenging since malignancy is a known risk factor for cardiovascular events [10]. There is a notable lack of large-scale real-world studies of colorectal cancer patients not treated with fluoropyrimidines as controls [11]. This gap in research limits our understanding of the true cardiovascular risk attributable to fluoropyrimidine treatment in cancer patients.

In this study, we report a territory-wide propensity score matched retrospective cohort study that evaluated the risk of cardiovascular events with fluoropyrimidine treatment using colorectal patients not treated with fluoropyrimidines as control. Additional comparison between 5-fluorouracil and capecitabine was performed to evaluate their cardiotoxicity profile. By evaluating the cardiovascular risk landscape associated with these widely used chemotherapeutic agents, our study aimed to inform clinical decision-making and optimize the safety and efficacy of colorectal cancer treatment.

## Method

### Study design

A retrospective cohort study was performed of adult patients diagnosed with colorectal carcinoma from January 1, 1993 to December 31, 2021 at public hospitals in Hong Kong. The primary objective was to compare cardiovascular outcome in those who received fluoropyrimidines (5-fluroracil or capecitabine) versus controls. The secondary objective was to determine which of the two fluoropyrimidine agents led to fewer adverse cardiovascular outcomes among patients with colorectal cancer. This study was approved by the institutional review board of the University of Hong Kong/ Hospital Authority Hong Kong West Cluster (UW 12–177). Need for patient consent was waived since the study involved only retrospective analysis of anonymized electronic health records.

### Data sources

Anonymized patient data were extracted from the Clinical Data Analysis and Reporting System (CDARS), an electronic health record system managed by the Hospital Authority that contains clinical data of all patients who receive healthcare services at public hospitals and clinics in Hong Kong. Demographic data including age and gender, diagnosis of colorectal carcinoma, prescription data of 5-fluroracil or capecitabine, as well as past medical history and clinical outcomes including hypertension, diabetes mellitus, chronic kidney disease, atrial fibrillation, myocardial infarction, ischemic stroke, heart failure hospitalization, deep vein thrombosis, pulmonary embolism, and mortality were extracted using International Statistical Classification of Diseases and Related Health Problems (ICD) codes listed in Supplementary Table 1.

### Outcomes

The primary outcome of the study was occurrence of major adverse cardiovascular event (MACE), a composite of myocardial infarction, ischemic stroke, and cardiovascular death. Cardiovascular death was defined as occurrence of death within 30 days of any cardiovascular event. Key secondary endpoints included cardiovascular death, myocardial infarction, ischemic stroke, new-onset atrial fibrillation, and ventricular tachyarrhythmia including ventricular tachycardia and ventricular fibrillation.

### Statistical analysis

Continuous variables are presented as mean ± standard deviation (SD) or median [interquartile range (IQR)]. Categorical variables are presented as frequency and percentage. Comparisons between two groups were performed using student’s T test, Mann-Whitney U test, Fisher’s exact test, or Chi-square test. Propensity score matching was performed using R package “MatchIt” version 4.5.4. A standardized mean difference of ≤0.1 following propensity score matching was considered significantly balanced [12]. Survival analysis was performed using Kaplan Meier estimate and Cox proportional hazard ratio using R package “survival” version 3.5-5. Competing risk regression was performed using R package “riskRegression” version 2023.03.22. Survival curve for depicting competing risk was generated using R package “cmprsk” version 2.2–11. Subgroup analysis and forest plot generation were performed using R package “SubgrPlots” version 0.1.3. A p-value < 0.05 was considered statistically significant.

## Results

### Study population

A total of 51,888 patients with colorectal carcinoma were included in the analysis, after excluding patients who had early crossover between 5-fluroracil and capecitabine within 1 year (*n* = 13,727), and those who had insufficient follow up data or died within 60 days from date of diagnosis or fluoropyrimidine initiation (*n* = 23,655). Patients who received fluoropyrimidines had a lower proportion of females (42.3% vs. 46.0%, *p* < 0.001*), were younger (61.8+/-10.9 vs. 72+/-12.4, *p* < 0.001*), and had a lower proportion of cardiovascular comorbidities including hypertension, diabetes mellitus, advanced chronic kidney disease stage 3 to 5, myocardial infarction, ischemic stroke, heart failure hospitalization, and deep vein thrombosis (Table 1). Propensity score matching in a 1:1 ratio was performed using R package “MatchIt”. After matching, 21,216 patients remained with 10,608 patients in each group. The standardized mean difference of each matched parameter was ≤0.1, indicating both groups were balanced (Table 1). Peak carcinoembryonic antigen (CEA) level was higher in the fluoropyrimidines group than the control group during the subsequent follow up period (5.04 [2.84–33.4] µg/l and 2.8 [1.80–4.83] µg/l respectively (< 0.001*)).

**Table 1:** Baseline characteristics before and after propensity score matching for control versus 5-flurouracil and capecitabine

Before propensity score matching						After propensity score matching			
	Combined *n* = 51,888	Control *n* = 41,274	5-flurouracil & capecitabine *n* = 10,614	SMD	*p* value	Combined *n* = 21,216	Control *n* = 10,608	5-flurouracil & capecitabine *n* = 10,608	SMD
Sex(female), n(%)	2,347(45.2%)	18,984(46%)	4,492(42.3%)	-0.07	< 0.001*	8,794(41.4%)	4,302(40.6%)	4,492(42.3%)	0.04
Age(years), mean ± SD	69.9+/-12.8	72+/-12.4	61.8+/-10.9	-0.94	< 0.001*	61.9+/-11.2	62+/-11.6	61.8+/-10.9	-0.02
Hypertension, n(%)	18,004(34.7%)	14,733(35.7%)	3,271(30.8%)	-0.11	0.001*	6,416(30.2%)	3,145(29.6%)	3,271(30.8%)	0.03
Diabetes mellitus, n(%)	9,012(17.4%)	7,430(18%)	1,582(14.9%)	-0.09	< 0.001*	3,151(14.9%)	1,569(14.8%)	1,582(14.9%)	0.01
Chronic kidney disease, n(%)	987(1.90%)	972(2.35%)	15(0.14%)	-0.59	< 0.001*	25(0.12%)	10(0.09%)	15(0.14%)	0.01
Atrial fibrillation, n(%)	2,474(4.77%)	2,281(5.53%)	193(1.82%)	-0.28	< 0.001*	310(1.46%)	117(1.10%)	193(1.82%)	0.05
Myocardial infarction, n(%)^#^	1,033(1.99%)	942(2.28%)	91(0.86%)	-0.15	< 0.001*	148(0.70%)	57(0.54%)	91(0.86%)	0.03
Ischemic stroke, n(%)	2,917(5.62%)	2,699(6.54%)	218(2.05%)	-0.32	< 0.001*	346(1.63%)	129(1.22%)	217(2.05%)	0.06
Heart failure hospitalization, n(%)	2,137(4.12%)	2,062(5%)	75(0.71%)	-0.51	< 0.001*	112(0.52%)	37(0.35%)	75(0.71%)	0.04
Deep vein thrombosis, n(%)	508(0.97%)	450(1.09%)	58(0.55%)	-0.07	< 0.001*	103(0.48%)	45(0.42%)	58(0.55%)	0.02
Pulmonary embolism, n(%)	244(0.47%)	199(0.482%)	45(0.42%)	-0.01	0.47	85(0.40%)	40(0.38%)	45(0.42%)	0.01

Asterisk (*) indicates statistically significant with p-value < 0.05. Standardized mean difference (SMD) < 0.1 after propensity score matching is considered sufficient balanced

### Cardiovascular outcome

A total of 18,801 patient-years of follow up were analyzed to evaluate cardiovascular safety of fluoropyrimidine use (9,484 patient-years in the fluoropyrimidines group and 9,318 patient-years in the control group). A major adverse cardiovascular event (MACE) occurred in 1.06% of patients and comprised cardiovascular death, non-fatal myocardial infarction, and non-fatal ischemic stroke. There was no significant difference in MACE risk between the two groups (HR 0.91, 95% confidence interval (95% CI): 0.70–1.18, *p* = 0.46). Cardiovascular death occurred in 0.41% of patients with no between group differences (HR 1.05, 95% CI: 0.69–1.60, *p* = 0.82).

The risk of developing myocardial infarction was similar in both groups (HR 0.73, 95% CI: 0.43–1.25, *p* = 0.25). During the 60 days before onset of follow up, 45 (0.42%) and 36 (0.34%) patients were labelled as having chest pain in the fluoropyrimidines group and control group respectively (*p* = 0.37). During the follow up period, only one patient developed myocardial infarction, with no statistically significant difference between fluoropyrimidines and control groups observed, partly owing to the small number of patients in this subgroup.

Peak troponin T level was higher in the fluoropyrimidines group than control group at 3.07 [1.74–4.39] µg/l and 0.10 [0.03–1.03] µg/l respectively (*p* = 0.02*). On the contrary, peak troponin I level was lower in the fluoropyrimidines group at 2.22 [0.62–8.10] µg/l and 5.45 [1.37–22.8] µg/l respectively (*p* = 0.008*). Given these contradictory findings, no definitive conclusion could be drawn about the relationship between fluoropyrimidine use and cardiac troponin level during myocardial infarction.

**Fig. 1:**
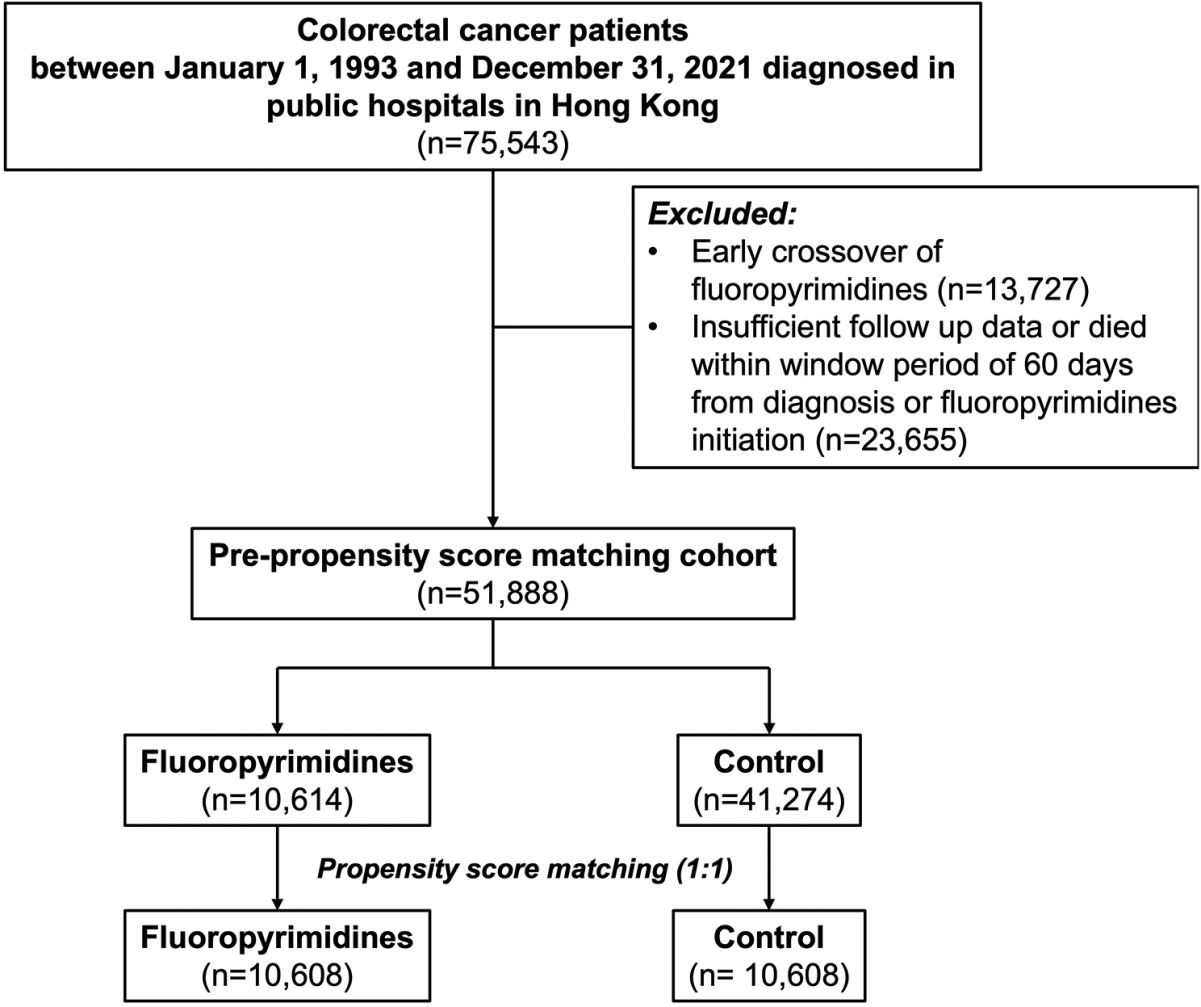
Cohort selection. A total of 75,543 patients with colorectal cancer diagnosed between January 1, 1993 and December 31, 2021 at public hospitals in Hong Kong were selected. After excluding those who had early crossover between 5-fluroracil and capecitabine within 1 year (*n* = 13,727), and those who had insufficient follow up data or died within 60 days from the date of diagnosis or fluoropyrimidine initiation (*n* = 23,655), 51,888 patients were included in the analysis. Among the 51,888 patients, 10,614 were treated with fluoropyrimidines. After propensity score matching in a 1:1 ratio, there remained 21,216 patients with 10,608 in each group

**Fig. 2:**
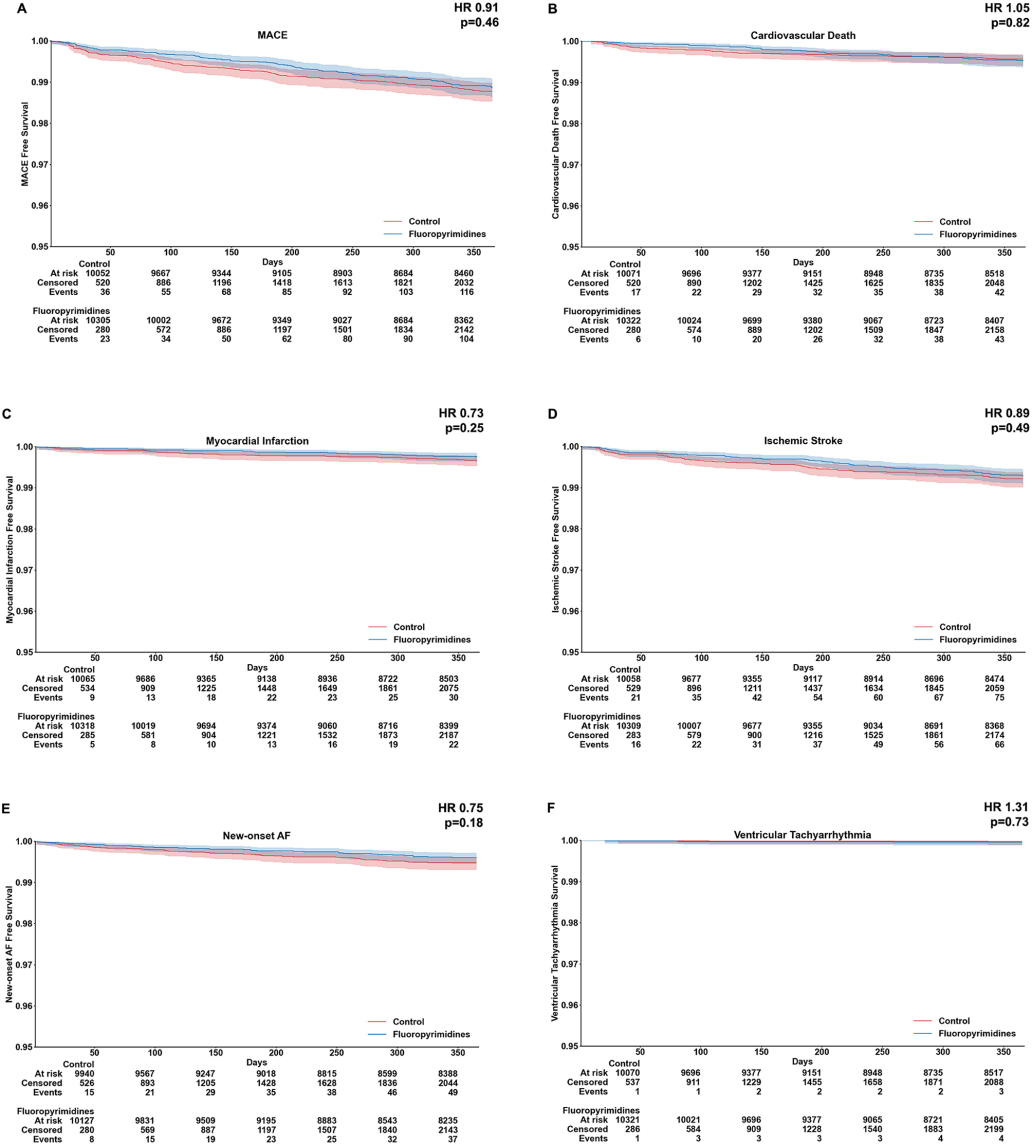
Kaplan Meier curve of cardiovascular events. There was no significant difference between fluoropyrimidines and controls in the incidence of (**A**) major adverse cardiovascular events (MACE) (HR 0.91, 95% CI: 0.70–1.18, *p* = 0.46), (**B**) cardiovascular death (HR 1.05, 95% CI: 0.69–1.60, *p* = 0.82), (**C**) myocardial infarction (HR 0.73, 95% CI: 0.50–1.25, *p* = 0.25), (**D**) ischemic stroke (HR 0.89, 95% CI: 0.64–1.24, *p* = 0.49), (**E**) new-onset atrial fibrillation (HR 0.75, 95% CI: 0.49–1.14, *p* = 0.18), or (**F**) ventricular tachyarrhythmia (HR 1.31, 95% CI 0.29–5.84, *p* = 0.73). **Abbreviation**: Hazard ratio, HR; 95% confidence interval, 95% CI

Finally, there was no difference in risk of developing ischemic stroke (HR 0.89, 95% CI: 0.64–1.24, *p* = 0.49), new-onset atrial fibrillation (HR 0.75, 95% CI 0.49–1.14, *p* = 0.18), or ventricular tachyarrhythmia (HR 1.31, 95% CI 0.29–5.84, *p* = 0.73) (Table 2; Figs. 1 and 2).

**Table 2:** Cardiovascular outcome for controls versus 5-flurouracil and capecitabine

				Cox proportional hazard Model		Competing risk Regression	
Variables	Combined *N* = 21,216	Control *n* = 10,608	5-flurouracil & capecitabine *n* = 10,608	Hazard Ratio (95% C.I.)	*P*-Value	Hazard Ratio (95% C.I.)	*P*-Value
Death	3,500(16.5%)	1837(17.3%)	1,663(15.7%)	0.89(0.83–0.95)	< 0.001*	-	-
MACE	225(1.06%)	117(1.10%)	108(1.02%)	0.91(0.70–1.18)	0.46	-	-
Cardiovascular Death	87(0.41%)	42(0.39%)	45(0.42%)	1.05(0.69–1.60)	0.82	-	-
Myocardial infarction	54(0.255%)	31(0.29%)	23(0.22%)	0.73(0.50–1.25)	0.25	0.75(0.44–1.29)	0.30
Ischemic stroke	143(0.674%)	75(0.71%)	68(0.64%)	0.89(0.64–1.24)	0.49	0.92(0.66–1.28)	0.61
New-onset Atrial fibrillation^#^	86/20,896 (0.41%)^#^	49/10,481 (0.47%)^#^	37/10,415 (0.36%)^#^	0.75(0.49–1.14)	0.18	0.77(0.50–1.18)	0.23
Ventricular tachycardia or fibrillation	7(0.03%)	3(0.03%)	4(0.04%)	1.31(0.29–5.84)	0.73	1.35(0.30–6.02)	0.69

^#^ New-onset atrial fibrillation was analyzed only in patients without known atrial fibrillation. Asterisk (*) indicates statistically significant with p-value < 0.05. Major adverse cardiovascular event (MACE) includes myocardial infarction, ischemic stroke, and cardiovascular mortality. Venous thromboembolism includes deep vein thrombosis and pulmonary embolism

### Competing risk regression

To minimize the effect of competing risk from the relatively high all-cause mortality in this cohort of cancer patients we further analyzed the cardiovascular outcomes between our treatment and matched control groups using a competing risk regression model. Competing risk regression to adjust the competing effect of all-cause mortality was performed using R package “riskRegression”. Consistent with previous findings, no increased risk for myocardial infarction (*p* = 0.30), ischemic stroke (*p* = 0.61), new-onset atrial fibrillation (*p* = 0.23), or ventricular tachyarrhythmia (*p* = 0.69) was observed (Table 2).

### Subgroup analysis

**Fig. 3:**
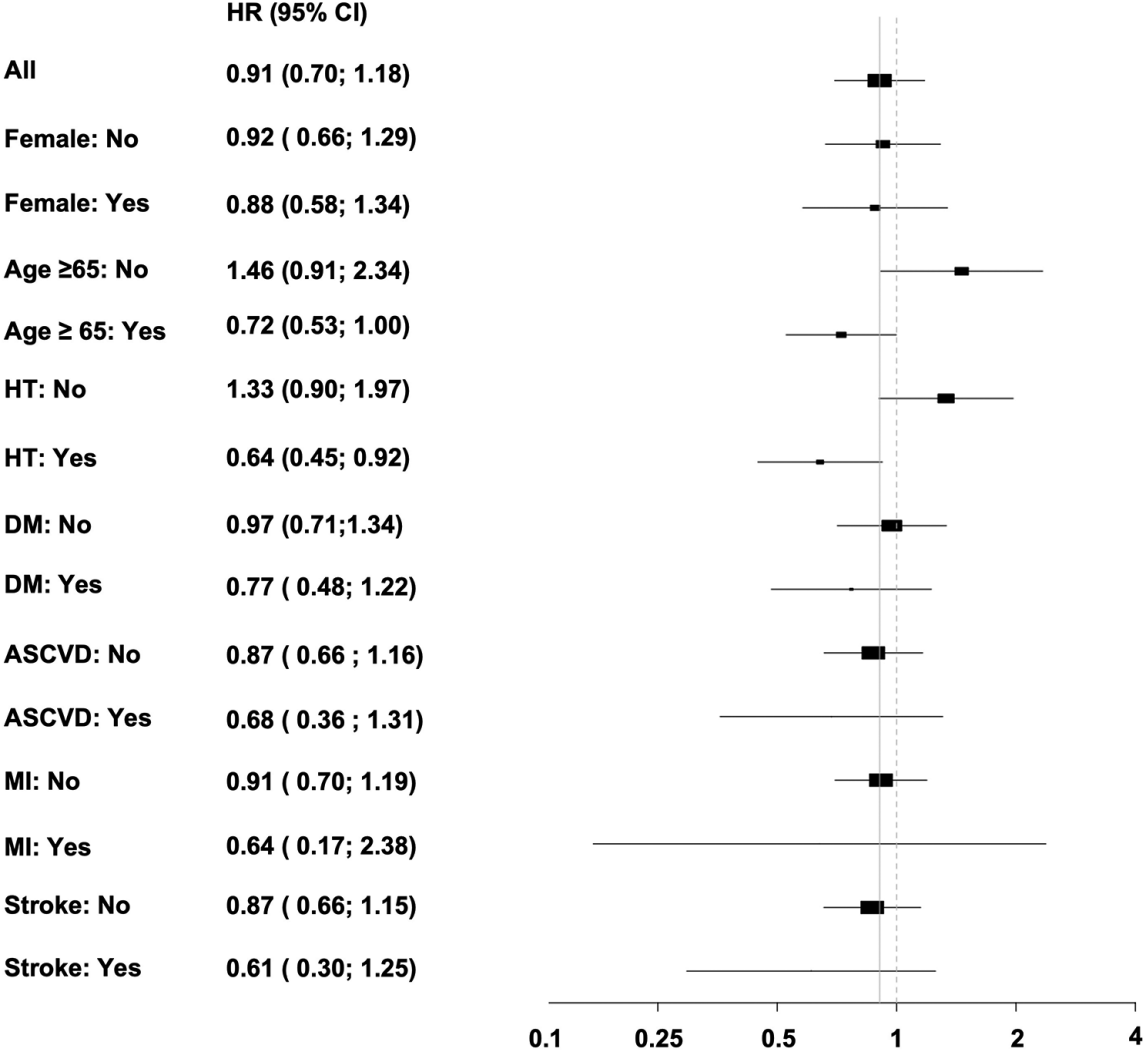
Subgroup analysis for major adverse cardiovascular event (MACE). The forest plot shows hazard ratio for MACE in seven high-risk groups, including male sex, age ≥ 65 years, hypertension (HT), diabetes mellitus (DM), atherosclerotic cardiovascular disease (ASCVD), myocardial infarction (M), andr stroke. None of the high-risk subgroups analyzed were found to have a significantly elevated risk of MACE during fluoropyrimidine use. Abbreviation: Hazard ratio, HR; 95% confidence interval, 95% CI

One commonly encountered clinical question is whether it is safe to prescribe fluoropyrimidines to patients with known cardiovascular disease. Subgroup analysis was performed to study the effect of fluoropyrimidines among high-risk patient groups. Figure 3 is a forest plot showing the hazard ratio for MACE in various high-risk groups, including male sex, age > = 65 years, hypertension, diabetes mellitus, and history of myocardial infarction or ischemic stroke. None of the high-risk subgroups analyzed were found to significantly elevate the risk of MACE during fluoropyrimidine use (Fig. 3).

### Predictors of major adverse cardiovascular events (MACE)

We also investigated the potential predictors of major adverse cardiovascular events in adult patients with colorectal carcinoma. We demonstrated that history of ischemic stroke was the strongest predictor of MACE amongst all the tested factors (HR 10.4, 95% C.I. 7.06–15.2, *P* < 0.001*). This was followed by chronic kidney disease (HR 9.04, 95% C.I. 5.35–15.3, *p* < 0.001*), heart failure hospitalization (HR 5.88, 95% C.I. 2.61–13.2, *p* < 0.001*) and myocardial infarction (HR 5.51, 95% C.I. 2.83–10.7, *p* < 0.001*). Other predictors including deep vein thrombosis (HR 3.85, 95% C.I. 1.43–10.3, *p* = 0.008*), pulmonary embolism (HR 3.76, C.I. 1.2–11.8, *p* = 0.02*), age > 65 years (HR 3.07, 95% C.I. 2.32–4.06, *p* < 0.001*), diabetes mellitus (HR 2.78, 95% C.I. 2.1–3.67, *p* < 0.001*), hypertension (HR 2.77, 95% C.I. 2.13–3.6, *p* < 0.001*), and atrial fibrillation (HR 2.76, 95% C.I. 1.42–5.38, *p* = 0.003*), were also associated with increased risk of developing MACE. It is worth noting that in our cohort, use of 5-flurouracil and capecitabine (HR 0.91, 95% C.I. 0.70–1.18, *p* = 0.46), as well as male sex (HR 1.14, 95% C.I. 0.87–1.49, *p* = 0.33) had no significant predictive value for the development of MACE (Table 3).

**Table 3:** Predictors of major adverse cardiovascular events (MACE)

Predictors	Hazard ratio (95% C.I.)	*p*-value
5-flurouracil & capecitabine	0.91(0.70–1.18)	0.46
Male	1.14(0.87–1.49)	0.33
Age > 65 years	3.07(2.32–4.06)	< 0.001*
Hypertension	2.77(2.13–3.60)	< 0.001*
Diabetes mellitus	2.78(2.10–3.67)	< 0.001*
Chronic kidney disease	9.04(5.35–15.3)	< 0.001*
Atrial fibrillation	2.76(1.42–5.38)	0.003*
Myocardial infarction	5.51(2.83–10.7)	< 0.001*
Ischemic stroke	10.4(7.06–15.2)	< 0.001*
Heart failure hospitalization	5.88(2.61–13.2)	< 0.001*
Deep vein thrombosis	3.85(1.43–10.3)	0.008*
Pulmonary embolism	3.76(1.20–11.8)	0.02*

### 5-flurouracil versus capecitabine

A previous prospective study in 527 patients suggested that 5-flurouracil was associated with lower risk of cardiotoxicity than capecitabine [5]. There has subsequently been a lack of real-world data to validate this finding. We utilized our large cohort of patients to determine whether a similar phenomenon was evident. Among the 10,614 patients who received fluoropyrimidines for colorectal cancer, 7,366 received capecitabine and 3,248 received 5-fluroracil. After 1:1 propensity score matching, 3,243 patients with 5-flurouracil and the same number of patients with capecitabine were analyzed. All baseline conditions were balanced with standardized mean differences < 0.1 Details of patient characteristics before and after matching are listed in Table 4.

**Table 4:** Baseline characteristics before and after propensity score matching for 5-flurouracil versus capecitabine

Before propensity score matching						After Propensity Score Matching			
Baseline conditions	Combined *N* = 10,614	5-flurouracil *n* = 7,366	capecitabine *n* = 3,248	SMD	*p* value	Combined *N* = 6,486	5-flurouracil *n* = 3,243	capecitabine *n* = 3,243	SMD
Sex (female), n(%)	4,492(42.3%)	3,160(42.9%)	1,332(41.0%)	-0.37	0.07	2,645(40.8%)	1,313(40.5%)	1,332(41.1%)	0.01
Age (years), mean ± SD	61.8+/-10.9	62.9+/-10.7	59.2+/-10.9	-0.34	< 0.001*	59.2+/-11.1	59.3+/-11.2	59.2+/-10.9	-0.01
Hypertension, n(%)	3,271(30.8%)	2,601(35.3%)	670(20.6%)	-0.36	< 0.001*	1,300(20.0%)	630(19.4%)	670(20.7%)	0.03
Diabetes mellitus n(%)	1,582(14.9%)	1,228(16.7%)	354(10.9%)	-0.18	< 0.001*	712(11.0%)	358(11.0%)	354(10.9%)	-0.00
Chronic kidney disease, n(%)	15(0.14%)	1(0.01%)	14(0.43%)	0.06	< 0.001*	15(0.23%)	1(0.03%)	14(0.43%)	0.06
Atrial fibrillation n(%)	193(1.82%)	148(2.01%)	45(1.39%)	-0.05	0.02*	92(1.42%)	47(1.45%)	45(1.39%)	-0.01
Myocardial infarction n(%)^#^	91(0.86%)	75(1.02%)	16(0.49%)	-0.07	0.008*	36(0.56%)	20(0.62%)	16(0.49%)	-0.03
Ischemic stroke n(%)	218(2.05%)	176(2.39%)	42(1.29%)	-0.10	< 0.001*	78(1.20%)	36(1.11%)	42(1.30%)	0.02
Heart failure hospitalization n(%)	75(0.71%)	53(0.72%)	22(0.68%)	-0.01	0.90	43(0.66%)	21(0.65%)	22(0.68%)	0.00
Deep vein thrombosis n(%)	58(0.55%)	38(0.52%)	20(0.62%)	0.01	0.57	36(0.56%)	16(0.49%)	20(0.62%)	0.02
Pulmonary embolism n(%)	45(0.42%)	34(0.46%)	11(0.34%)	-0.02	0.42	27(0.42%)	16(0.49%)	11(0.34%)	-0.03

Asterisk (*) indicates statistically significant with p-value < 0.05. Standardized mean difference (SMD) < 0.1 after propensity score matching is considered sufficient balanced

The proportion of patients who developed MACE during the follow up period was similar: 0.80% and 0.89% in 5-fluroracil and capecitabine groups respectively (HR 1.09, 95% CI: 0.64–1.85, *p* = 0.76). There was also no evidence of elevated risk for cardiovascular death, myocardial infarction, ischemic stroke, new-onset atrial fibrillation, or ventricular tachyarrhythmia in either group. Competing risk regression was performed and even after adjusting for all-cause mortality the findings were consistent on standard Cox regression. (Table 5).

**Table 5:** Cardiovascular outcome for 5-flurouracil versus capecitabine

				Cox proportional hazard model		Competing risk Regression	
Variables	Combined *N* = 6,486	5-flurouracil *n* = 3243	capecitabine *n* = 3243	Hazard Ratio (95% C.I)	*P*-Value	Hazard Ratio (95% C.I.)	*P*-Value
Death	1,055(16.3%)	442(13.6%)	613(18.9%)	1.35(1.2–1.53)	< 0.001*	-	-
MACE	55(0.85%)	26(0.80%)	29(0.89%)	1.09(0.64–1.85)	0.75	-	-
Cardiovascular Death	25(0.39%)	14(0.43%)	11(0.34%)	0.77(0.35–1.69)	0.51	-	-
Myocardial infarction	13(0.20%)	8(0.25%)	5(0.15%)	0.61(0.20–1.87)	0.39	0.60(0.20–1.83)	0.39
Ischemic stroke	35(0.54%)	12(0.37%)	23(0.71%)	1.87(0.93–3.76)	0.08	1.87(0.93–3.76)	0.08
New-onset Atrial fibrillation^#^	22/6,394(0.34%)^#^	9/3,196(0.28%)^#^	13/3,198(0.41%)^#^	1.41(0.60–3.30)	0.43	1.40(0.60–3.26)	0.44
Ventricular tachycardia or fibrillation	3(0.05%)	1(0.03%)	2(0.06%)	1.96(0.18–21.7)	0.58	1.95(0.18–21.2)	0.58

^#^ New-onset atrial fibrillation was analyzed only in patients without known atrial fibrillation. Asterisk (*) indicates statistically significant with p-value < 0.05. Major adverse cardiovascular event (MACE) includes myocardial infarction, ischemic stroke, and cardiovascular mortality. Venous thromboembolism includes deep vein thrombosis and pulmonary embolism

## Discussion

In this large-scale territory-wide propensity score matched retrospective cohort study involving 21,216 patients with colorectal cancer and those not treated with fluropyrimidines serving as controls, we demonstrated that fluoropyrimidine did not increase the risk of a major adverse cardiovascular event (MACE), cardiovascular death, or other specific cardiovascular conditions. (Graphical Abstract) There was also no significant difference in the risk of cardiovascular events between the 5-fluorouracil and capecitabine groups. These findings remained consistent after adjusting for all-cause mortality in a competing risk regression analysis.

### Mechanism of cardiotoxicity

The pathophysiology of fluoropyrimidine-induced cardiotoxicity has not been fully elucidated. Several potential mechanisms such as coronary vasospasm, endothelial injury, and accumulation of toxic metabolites have been proposed. Coronary artery vasospasm has been observed during coronary angiography in patients who presented with chest pain after receiving 5-fluorouracil [13, 14]. The absence of vascular occlusions in most patients does not support arterial thrombosis as the primary mechanism of cardiotoxicity [15–18]. Mechanistically, animal models demonstrated that 5-fluorouracil induces vasoconstriction via protein kinase C pathways [19]. 

### Fluoropyrimidine cardiotoxicity

Common clinical manifestations of fluropyrimidine cardiotoxicity include chest pain, dyspnea, palpitations, and electrocardiography (ECG) changes [20–22]. Its incidence varies from 1 to 35% according to different studies, with most series’ reporting a risk of around 8% [20, 23–25]. These manifestations are usually reversible with supportive care and discontinuation of associated chemotherapy [26–28]. Major cardiovascular events such as myocardial infarction are less commonly encountered with a reported incidence of 0.9% among gastrointestinal cancer patients treated with fluorouracil without prior ischemic heart disease [9]. There is a notable lack of large-scale real-world studies in patients with colorectal cancer and those not treated with fluoropyrimidines serving as controls [11]. Our currently reported propensity score matched cohort study provides the most real-world data to date with colorectal patients not treated with fluropyrimidines serving as controls. Fluoropyrimidine did not increase the risk of a major adverse cardiovascular event (MACE), cardiovascular death, or other specific cardiovascular conditions in such patients. The occurrence of cardiovascular events was driven mainly by traditional cardiovascular risk factors such as age, sex, hypertension, diabetes mellitus, and known atherosclerotic cardiovascular disease, not use of fluropyrimidines.

### 5-fluorouracil vs. capecitabine

Capecitabine is an orally administered prodrug of 5-fluorouracil. It is activated in tumors and the liver via a three-step enzymatic process and often used as a replacement for 5-fluorouracil in many chemotherapeutic regimens due to its similar efficacy, easier administration, and more favorable toxicity profile. Nonetheless which of 5-fluorouracil or capecitabine is more cardiotoxic remains a topic of debate. A prospective analysis indicated a lower incidence of cardiotoxicity with capecitabine [27], while other studies have reported contradictory findings [5, 21]. To provide data for this important clinical knowledge gap, 6,486 propensity score-matched patients treated with either 5-fluorouracil or capecitabine were analyzed as a sub-study. The two chemotherapeutic agents did not produce significant differences in terms of major cardiovascular events.

### Clinical implications

Given the high prevalence of both cardiovascular disease and colorectal cancer, the choice between 5-fluorouracil and capecitabine, from a cardiotoxicity perspective, carries clinical significance. In our study, we demonstrated that the use of fluoropyrimidines did not increase the risk of major adverse cardiovascular events (MACE) in patients with colorectal cancer. Furthermore, there was no clinically significant difference in terms of MACE between 5-fluoruracil and capecitabine.

### Limitations

This study has several limitations. First, its retrospective and observational nature may have resulted in more residual confounding and bias than a prospective randomized study. Nonetheless crucial confounding variables like age and pre-existing cardiovascular conditions were adjusted. Second, since our study population comprised patients of predominantly Chinese ethnicity, it is more difficult to generalize our conclusions to other populations and ethnicities.

## Conclusion

In this large-scale territory-wide propensity score matched retrospective cohort study, fluoropyrimidine use did not increase the risk of major adverse cardiovascular events (MACE). There was also no significant difference in the risk of cardiovascular event occurrence between 5-fluorouracil and capecitabine.

## Electronic supplementary material

Below is the link to the electronic supplementary material.

Supplementary Material 1

## Data Availability

No datasets were generated or analysed during the current study.

## Abbreviations

ICD: International statistical classification of diseases and related health problems
MACE: Major adverse cardiovascular outcome
