# Supplementary material for: Cardiovascular safety of 5-fluorouracil and capecitabine in colorectal cancer patients: real-world evidence
Source: Cardiooncology. 2025 Jan 15;11:3. doi: 10.1186/s40959-024-00294-2 (PMC11734475; doi:10.1186/s40959-024-00294-2)
Supplement: Supplementary file 1 — Supplementary Material 1 [file 40959_2024_294_MOESM1_ESM.docx]

**SUPPLEMENTARY APPENDIX**

**Supplementary Table S1.** International Classification of Diseases, Ninth Revision, Clinical Modification (ICD-9-CM) and International Classification of Primary Care (ICPC) codes

| **Diagnosis** | **ICD-9-CM and ICPC codes** |
| --- | --- |
| Atrial fibrillation | 427.3 |
| Diabetes mellitus | 250, T89*, T90* |
| Deep vein thrombosis | 453.8 |
| Hypertension | 401, 405, K86*, K89* |
| Heart failure | 429 |
| Ischemic stroke and transient ischemic attack | 433, 434, 435, 436 |
| Myocardial infarction | 410 |
| Pulmonary embolism | 415.1 |
| Ventricular tachycardia and fibrillation | 427 |

Asterisk (*) indicates ICPC codes
